# A review of the microgastropod genus *Systenostoma* Bavay & Dautzenberg, 1908 and a new subterranean species from China (Gastropoda, Pulmonata, Hypselostomatidae)

**DOI:** 10.3897/zookeys.410.7488

**Published:** 2014-05-20

**Authors:** Adrienne Jochum, Rajko Slapnik, Marian Kampschulte, Gunhild Martels, Markus Heneka, Barna Páll-Gergely

**Affiliations:** 1 Department of Community Ecology, Institute of Ecology and Evolution, Baltzerstrasse 6, University of Bern, CH-3012 Bern, Switzerland; 2 Drnovškova pot 2, Mekinje, 1240 Kamnik, Slovenia; 3 Universitätsklinikum Gießen und Marburg GmbH−Standort Gießen, Zentrum für Radiology, Abteilung für Radiologie, Klinik-Str. 33, 35385 Gießen, Germany; 4 RJL Micro & Analytic GmbH, Im Entenfang 11, 76689 Karlsdorf-Neuthard, Germany; 5 Department of Biology, Shinshu University, Matsumoto 390-8621, Japan

**Keywords:** Taxonomy, subterranean snail, Pupillidae, Vertiginidae, conservation, cave-dwelling species

## Abstract

A review of the microgastropod genus *Systenostoma* is provided. Thai and Malaysian species are transferred to a new genus, *Angustopila* (type species: *Systenostoma tamlod* Panha & Burch, 1999). A new subterranean *Angustopila* species is described here. Conchologically, the new species is most similar to the cave-dwelling, Thai *A. tamlod* (Panha & Burch, 1999). One Thai species (*Systenostoma edentata*) is transferred to the genus *Hypselostoma*. Vietnamese members remain in the genus *Tonkinospira* (nomen novum) for *Systenostoma* Bavay & Dautzenberg, 1908 (non *Systenostoma* Marsson, 1887). A comprehensive map of former *Systenostoma* species is presented. SEM and NanoCT images, including a video of *A. huoyani* **sp. n.** internal shell morphology, provide novel perspectives of the shells of *Angustopila* and of the scarcely known Vietnamese *Tonkinospira* species. The biology of these snails is not yet known but collection localities suggest a troglophilic ecology.

## Introduction

Microgastropods are less than 5 mm in size and represent the majority of worldwide tropical land snail diversity. They are restricted to specific microhabitats such as limestone rock surfaces or caves, have limited active dispersal ability and thus, frequently demonstrate high local endemism. Due to their small size and high degree of endemicity, our knowledge of the taxonomy and ecology of microgastropod taxa such as the hypselstomatid genus *Systenostoma* is limited. Consequently, very little is known about tiny species and thus, the complex systematics of most tropical microgastropod groups is based on conchological characters only.

The microgastropod genus *Systenostoma* was established as a subgenus of *Helix* by Bavay and Dautzenberg (1908) for two Vietnamese species described in the same paper, namely *Helix (Systenostoma) pulverea* and *Helix (Systenostoma) pauperrima*. The diagnosis of *Systenostoma* however, was given in another paper published a year later (Bavay and Dautzenberg 1909). The third Vietnamese species was described as *Systenostoma defixa* Bavay & Dautzenberg (1912). After these descriptions, *Systenostoma* remained in the dark for almost four decades. Jaeckel (1950) described *Angustopila depressa* from the debris of an unknown Tonkinese (Northern Vietnam) tropical river. Therefore, knowledge of the distribution and ecology of this species is lacking. More recently, four species of *Systenostoma* were described from Thailand, namely *Systenostoma concava* and *Systenostoma elevata* by Thompson and Upatham (1997) and *Systenostoma edentata* and *Systenostoma tamlod* by Panha and Burch (1999). The genus was also reported from Malaysia, but the systematic position of the Malaysian samples is not yet clarified. Although Benthem-Jutting’s (1949) figure of “*Hypselostoma laidlawi*” (Fig. 9) and her *Paraboysidia neglecta* (Benthem-Jutting, 1961) are similar and represent the same species according to her explanation, these illustrations likely show two different species (see Panha and Burch 1999).

The classification of *Systenostoma* is problematic. Together with the suspected, closely related genera (e.g. *Acinolaemus* Thompson & Upatham, 1997, *Anauchen* Pilsbry, 1917, *Boysidia* Ancey, 1881, *Hypselostoma* Benson, 1856, *Krobylos* Panha & Burch, 1999, *Gyliotrachela* Tomlin, 1930), *Systenostoma* is sometimes classified within Pupillidae (e. g. Panha and Burch 1999) or Vertiginidae (e.g. Thompson and Upatham 1997). These related genera are classified within Hypselostomatidae by Schileyko (1998). After examining the type species of the genus, Schileyko (1998) concluded that unlike these other genera, *Systenostoma* probably does not belong to Hypselostomatidae, but rather, likely belongs to Helicodiscidae because of the characteristic spiral sculpture. Later, Schileyko postulated that the genus is possibly related to *Aulacospira* as considered by Pilsbry (1917) or to *Pupisoma* (Valloniidae) (Schileyko 2011). Before the description of the Thai species, previous diagnoses (Bavay and Dautzenberg 1909, Pilsbry 1917, Zilch 1959) described the genus as a taxon lacking apertural dentition. Thompson and Upatham (1997) claimed that *Systenostoma* species bear no teeth and that a low fold may be present on the parietal wall. Moreover, they described the sculpture as “dense mesh of very fine granular reticulation superimposed upon which are fine spiral threads. Spiral sculpture may be present or absent on the protoconch”. Thompson and Upatham (1997) hypothesized a close relationship between *Systenostoma* and *Acinolaemus* based on the likely, homologous parietal lamella and similar protoconch sculpture.

All *Systenostoma* have a relatively simple shell compared to the other members of the family Hypselostomatidae, whose shells usually have oddly coiled shapes and multiple apertural denticles. Still, *Systenostoma* species show a high diversity in general shell shape, aperture shape and dentition and shell sculpture. *Systenostoma* seems to represent a “collection bin” taxon for species possessing few or no denticles. The reduction of the apertural teeth however, could have evolved independently in different evolutionary lineages. In this case, congeners may well have been classified/lumped within one genus but systematically belong to at least three genera. In the following, we describe a new, subterranean species from China, present an overview of all former *Systenostoma* species, describe a new genus for Thai, Malaysian and the new Chinese species, transfer *Systenostoma edentata* to the genus *Hypselostoma* and assign a new name (*Tonkinospira*) to the Vietnamese species because the name *Systenostoma* (non *Systenostoma* Marsson 1887) is preoccupied. We present SEM and Nano-CT images of shells of the new species and the scarcely known Vietnamese members of the genus.

## Material and methods

### Abbreviations

RBINS Royal Belgian Institute of Natural Sciences (Brussels, Belgium)

SMF Senckenberg Forschungsinstitut und Naturmuseum (Frankfurt am Main, Germany)

MCSMNH Malacological collection of the Slovenian Museum of Natural History (Ljubljana, Slovenia)

SMNS Staatliches Museum für Naturkunde Stuttgart (Stuttgart, Germany)

### Image acquisition

**SEM:** One paratype of *Angustopila huoyani* sp. n. was mounted on an aluminium stub, gold-palladium sputtered using the Edwards Kniese Sputter Coater S150B (Marburg, Germany) and subsequently scanned on the CamScan CS 24 scanning electron microscope (Dortmund, Germany). Specimens of *Tonkinospira* nom. n. were non-coated and imaged with the Zeiss EVO LS15 scanning electron microscope (Jena, Germany) using the Variable Pressure (VP) mode.

**Micro-CT:** *Tonkinospira* nom. n. species were imaged using a nano-computed tomography system (nano-CT), manufactured and developed by Bruker-Micro-CT/SkyScan (SkyScan 2011, Kontich, Belgium) at the Department of Experimental Radiology, Justus-Liebig University Biomedical Research Center Seltersberg (BFS), Giessen, Germany. The scanner is based on a nanofocus tube generating X-rays in cone-beam geometry. Briefly, the system contains an open pumped type X-ray source, a LaB6 cathode and a transmission anode consisting of a tungsten-coated beryllium window. Enhanced edge sharpness is gained by a high-focussed X-ray spot of 300 nm side length (see Langheinrich et al. (2010)) for more details). Specimens of *Tonkinospira* nom. n. were mounted on a computer-controlled stage. They were then scanned 180° around their vertical axis in rotation steps of 0.2° at 80 kV tube voltage and 120 µA tube current. Reconstruction of cross sectional images was performed using a modified Feldkamp cone-beam reconstruction algorithm. Image resolution of the cross sectional images was 1 µm isotropic voxel side length with a grey scale resolution of 8 bit. The video of *Angustopila huoyani* sp. n. was created using a SkyScan 1172 scanner at RJL Micro & Analytic GmbH, Karlsdorf-Neuthard, Germany. The scanner is equipped with a sealed micro focus x-ray source and a 11 Mpx CCD detector. The specimen was scanned with 4 µm voxel size in rotation steps of 0.6° at 59 kV tube voltage and 167 µA tube current. Reconstruction with cross sectional images followed the same aforementioned, cone-beam reconstruction algorithm. Image resolution of the cross sectional images was 4 µm isotropic voxel side length with a grey scale resolution of 8 bit. The animated video was generated using a direct volume rendering method implemented in the software CTvox.

**Digital images:** *Angustopila huoyani* sp. n. was photographed using a Kontron-Electronik-ProgRes-3012 microscope camera (Jena, Germany) and a Leitz MZ12 stereomicroscope.

## Taxonomy

### Family Hypselostomatidae Zilch 1959

#### Genus Angustopila

Jochum, Slapnik & Páll-Gergely gen. n.

http://zoobank.org/2DD2207C-8C0E-46F0-A1D3-12F480F105BD

http://species-id.net/wiki/Angustopila

##### Type species.

*Systenostoma tamlod* Panha & Burch, 1999.

##### Diagnosis.

*Angustopila* gen. n. is characterized by a very small, smooth, conical shell with regular, moderately increasing whorls. The body whorl sometimes extends beyond the penultimate whorl in profile. The sculpture of the protoconch is usually ornamented by spiral and radial lines resulting in a powdery, reticulated surface. The protoconch is slightly recessed into the second whorl. Aperture slightly or not adnate, with usually one or two denticles, peristome slightly reflexed.

##### Etymology.

The name derives from the combination of the Latin angustus (= narrow) and pila (= pillar, column). Gender: feminine.

##### Remarks.

*Angustopila* gen. n. differs from *Tonkinospira* nom. n. (former Vietnamese *Systenostoma*) by smaller shell size, more elevated spire, slightly reflexed apertural rim and general dentition present within the aperture. *Acinolaemus* usually has more teeth and a turban-like shell. *Krobylos* species have angulated whorls, lack spiral lines on the shell and possess a relatively large, toothless, adnate aperture.

##### Distribution.

The genus is known from Thailand and Malaysia. The Chinese *Angustopila huoyani* sp n. is located very distant, almost 1500 km from the northern Thai localities.

#### *Angustopila concava* (Thompson & Upatham, 1997)

*Systenostoma concava* Thompson & Upatham, 1997: Bulletin of the Florida Museum of Natural History, 39 (7): 231–232, Fig. 32–38. [“Thailand, Nakhon Ratchasima Province, limestone hill 3.4 km west of Ban Mu Si, 380 m altitude (14°32.0'N, 101°22.5'E)”]

*Systenostoma concava* – Panha and Burch 2005: Malacological Review, 37/38: 118–119, Fig. 101.

#### *Angustopila elevata* (Thompson & Upatham, 1997)

*Systenostoma elevata* Thompson & Upatham, 1997: Bulletin of the Florida Museum of Natural History, 39 (7): 232–233, Fig. 39–43. [“Thailand, Chiang Mae Province, Doi Chiang Dao (Mountain), 7 km west of Chiang Dao; 600 m altitude (19°24.3'N, 98°54.2'E)”]

*Systenostoma elevata* – Panha and Burch 2005: Malacological Review, 37/38: 120–121, Fig. 103.

#### Angustopila huoyani

Jochum, Slapnik & Páll-Gergely sp. n.

http://zoobank.org/2101F700-9723-422F-B70B-F8D0C2D20345

http://species-id.net/wiki/Angustopila_huoyani

Figure 4 –5 , Video 1

##### Type material.

China, Hunan, Longshan (龙山县), Huoyan (火焰), Feihu Dong (飞虎洞), (29°12.53'N, 109°18.37'E, 550 m alt.), soil, leg. Verovnik, 13.04.1997, MCSMNH 50312/1 (holotype), MCSMNH 50312/2–9 (8 paratypes).

##### Diagnosis.

A tiny, thin-shelled conical snail with very deep and narrow umbilicus, 5 shouldered whorls and two apertural denticles (parietal and palatal).

##### Description.

Shell thin, greyish white, semi opaque; conical, widest at its base, with a homogeneous powdery superficial texture and regularly increasing, shouldered whorls separated by deep suture; smooth with no notable spiral or radial sculpture. It is characterized by very fine irregular axial lamellae and reticulating microgranules producing the powdery superficial texture; protoconch shows reticulating granules and recognisable radial lines only at the upper part of the first whorl; aperture semi-circular, slightly oblique from ventral view; peristome very slightly thickened and reflexed; parietal callus adnate (attached to the penultimate whorl); aperture with two well-developed but short denticles, one on the parietal and the other on the palatal side; umbilicus very deep and narrow.

**Video 1.** Micro-CT Video of *Angustopila huoyani* sp. n. Video: Markus Heneka. http://www.pensoft.net/J_FILES/1/articles/7488/Jochum_Video_1.avi

##### Measurements.

See Table 1.

**Table 1.** Shell measurements (mm) for *Angustopila huoyani* sp. n. from the type locality. SH - shell height, SW - shell width, AH - aperture height, AW - aperture width, SW/SH×100 - shell width shared with shell height and multiplied 100, AW/AH×100 – aperture width shared with aperture height and multiplied 100).

SH	SW	AH	AW	SW/SH×100	AW/AH×100	specimen
1.09	0.87	0.33	0.43	80	133.33	Holotype
1.13	0.91	0.41	0.43	80.77	105.26	Paratype
1.09	0.89	0.35	0.41	82	118.75	Paratype
1.13	0.91	0.39	0.41	80.77	105.55	Paratype
1.20	0.96	0.43	0.43	80	100	Paratype
1.22	41641	0.39	0.46	83.93	116.66	Paratype
1.09	0.89	0.35	0.39	82	112.5	Paratype
1.09	0.91	0.33	0.39	84	120	Paratype
1.30	0.89	0.39	0.37	68.33	94.44	Paratype

**Table 2.** Average. minimum value (min). maximum value (max). variance of values (var) and standard deviation of a set of values (stdev) for *Angustopila huoyani* sp. n. N = 30.

	SH	SW	AH	AW	SW/SH×100	AW/AH×100
Average	1.17	0.94	0.37	0.43	80.09	114.20
Min	1.04	0.85	0.33	0.37	68.33	94.44
Max	1.30	1.04	0.43	0.46	87.50	133.33
Var	0.0058	0.0031	0.0008	0.0007	15.4080	79.0895
stdev	0.0765	0.0560	0.0288	0.0288	3.9253	8.8932

##### Differential diagnosis.

*Tonkinospira defixa*, *Tonkinospira pulverea*, *Tonkinospira pauperrima* and *Tonkinospira depressa* are much larger than the new species, have reticulated sculpture and lack denticles in the aperture. Moreover, *Tonkinospira defixa* has a more depressed spire, fewer whorls, wider umbilicus and slightly keeled body whorl; *Tonkinospira pulverea* has fewer whorls, a comparatively larger aperture, somewhat keeled, wider body whorl and its umbilicus is partly closed by the apertural margin; *Tonkinospira pauperrima* shows increased bulging in whorl configuration; *Tonkinospira depressa* has a lower spire, slightly keeled body whorl and a large aperture without denticles. *Hypselostoma* (?) *edentata* also lacks denticles in the aperture and possesses a very wide, laterally compressed body whorl. *Angustopila tamlod*, the most similar species, is slightly smaller, has fewer whorls, wider umbilicus and obvious spiral striation on the teleoconch. *Angustopila concava* has a much wider body whorl than that of *Angustopila huoyani*, has weaker apertural denticles and prominent spiral sculpture. *Angustopila elevata* has no denticles in the aperture and possesses a wider umbilicus and spirally striated shell. *Angustopila neglecta* (see also notes under that species) has a wider umbilicus and more rapidly growing whorls, resulting in a comparatively wider body whorl than in the new species. *Angustopila neglecta* also has spiral lines on the shell and its sinulus is wider. Shell characters and ecological information of all *Angustopila* species are presented in Table 3.

**Table 3.** The most important morphological traits and ecological information for *Angustopila huoyani* sp. n. and its congeners extracted from the literature.

	*Angustopila concava*	*Angustopila elevata*	*Angustopila huoyani* sp. n.	*Angustopila neglecta*	*Angustopila tamlod*
**Shell colour**	greyish white	light gray	greyish white	white	white
**Teleoconch sculpture**	spiral threads; weak growth wrinkles	spiral threads; weak growth wrinkles	very fine irregular axial lamellae, reticulating microgranules	spiral threads; growth wrinkles	spiral threads
**Protoconch sculpture**	spiral/ reticulated	reticulating granules	reticulating granules	not described	not decribed
**Aperture shape/ peristome**	kidney-shaped, oblique	ovate, oblique	semi-circular, oblique	ovate	semi-circular
**Aperture dentition**	angular, upper palatal, parietal	dentition lacking	parietal, palatal	angular, upper palatal, weak basal	parietal, palatal
**Umbilicus**	narrow	narrow	very narrow	relatively wide	very narrow
**Whorl number**	4.6–5.3	4.2–4.3	5.3	4.5	4.75
**Shell height (mm)**	1.02–1.21	0.92–0.99	1.04–1.3	1.2–1.8	0.9–1.0
**Ecology**	leaf litter & limestone talus	leaf litter & limestone talus	cave	cave (?)	cave

##### Etymology.

The new species is named after the Gorges of Huoyan, where the type locality is located.

##### Distribution.

The new species is known from the type locality only.

##### Ecology.

The new species is known only from the Feihu Dong (“Cave of the Wind Tiger”). *Angustopila huoyani* were culled from samples of rocky-loamy substrate collected in the entrance corridor of the cave. It is highly likely that the distribution of *Angustopila huoyani* sp. n. is restricted to this cave only.

##### Conservational status.

Our knowledge of the biogeography of the genus is very limited. However, we assume that most *Angustopila*, especially the cave-dwelling species, are narrow-range endemics. Since extreme endemism always makes species vulnerable to human encroachment, this species warrants conservation priority. Currently, no direct threats are known.

#### Angustopila neglecta

(van Benthem-Jutting, 1961)

Hypselostoma laidlawi – Benthem Jutting ? 1949: Bulletin of the Raffles Museum, 21: 19, Fig. 9.
Paraboysidia neglecta van Benthem Jutting 1961: Bulletin of the Raffles Museum, 26: 36, Plate 8, Fig. 2a. [“Biserat Caves, State of Jalor” and “Gua Che Yatin, Ulu Tembeling, Pahang”]
Hypselostoma laidlawi (referring to the figure in Benthem-Jutting (1949) as probably *Systenostoma* species) – Panha and Burch 1999: Walkerana, 10 (24): 125.

##### Remarks.

Although the specimen on Benthem Jutting’s (1949) figure is similar, it probably is not conspecific to her other figure (Benthem Jutting 1961). See detailed explanation in Panha and Burch (1999).

#### *Angustopila tamlod* (Panha & Burch, 1999)

*Systenostoma tamlod* Panha & Burch, 1999: Walkerana 10 (24): 118–121, Fig. 3. [“Lod Cave, Pang Ma Pa District, Mae Hong Son Province, 19°29'36"N, 98°17'18"E and 10°34'03"N, 98°16'41"E, 800 meters elevation (CUIZM, Ver 025), Thailand. All specimens were collected inside the cave, almost two kilometres from the entrance.”]

#### Genus Hypselostoma

Benson 1856

http://species-id.net/wiki/Hypselostoma

Hypselostoma Benson 1856b, The Annals and Magazine of Natural History, ser. 2, no. 17: 342. (nom. n. for *Tanystoma*Benson 1856a, non Latreille, 1829).

##### Type species.

*Tanystoma tuberiferum* Benson, 1856 by monotypy.

##### Remarks.

*Systenostoma edentata* Panha & Burch, 1999 differs from all former, Thai *Systenostoma* species by the relatively large, toothless, adnate aperture. It is probably a toothless member of a hypselostomatid genus other than former *Systenostoma*. Here, *Systenostoma edentata* is placed within the genus *Hypselostoma* because its similarity with *Hypselostoma panhai* Burch & Tongkerd, 2002. *Hypselostoma panhai* is not a typical member of the genus *Hypselostoma* in terms of shell characters, but was placed into this genus based on molecular data of Tongkerd et al. (2004).

#### *Hypselostoma* (?) *edentata* (Panha & Burch, 1999)

*Systenostoma edentatum* Panha & Burch, 1999: Walkerana, 10 (24): 121–124, Fig. 4a–d. [“Tamphatai National Park, Phrae Province, 18°36"20'N, 99°53"49'E, 650 meters elevation (CUIZM, Ver 022), Thailand”]

*Systenostoma edentata* – Panha and Burch 2005: Malacological Review, 37/38: 119–120, Fig. 102.

#### Genus Tonkinospira

Jochum, Slapnik & Páll-Gergely nom. n.

Systenostoma Bavay & Dautzenberg, 1908: Journal de Conchyliologie, 56: 243.
Systenostoma – Bavay and Dautzenberg 1909: Journal de Conchyliologie, 57: 196. (diagnosis).

##### Remarks.

The name *Systenostoma* Bavay & Dautzenberg, 1908 is preoccupied (non *Systenostoma* Marsson 1887, Bryozoa). Therefore, *Tonkinospira* Jochum, Slapnik & Páll-Gergely nom. n. is proposed as replacement.

##### Type species.

*Helix (Systenostoma) pauperrima* Bavay & Dautzenberg, 1908 by subsequent designation (Pilsbry 1917).

##### Diagnosis.

A genus of small, conical or depressed-conical species with regularly growing, rounded or angulated whorls. The sculpture is characterized by spiral lines on both the protoconch and the teleoconch, decussated by irregular radial lines resulting in a reticulated surface structure. The aperture is toothless, adnate or slightly adnate and shows a sharp peristome.

##### Etymology.

The new name is established by the fusion of Tonkin (northern Vietnam, the area of distribution) and the Latin spira (a coil, twist). Gender: feminine.

##### Remarks.

*Tonkinospira* differs from *Krobylos* by the increased degree of angulation of the whorls and the lack of spiral lines on the teleoconch. For differences with *Angustopila* gen. n., see above.

The systematic position of the genus is questionable. It most probably belongs to the family Hypselostomatidae, but its relationship with other families such as Helicodiscidae (see Schileyko 1998) or Valloniidae (see Schileyko 2011) cannot be excluded.

##### Distribution.

So far, the genus is reported from Northern Vietnam only.

#### Tonkinospira defixa

(Bavay & Dautzenberg, 1912)

http://species-id.net/wiki/Tonkinospira_defixa

Figure 1

Systenostoma defixa Bavay & Dautzenberg, 1912: Journal de Conchyliologie, 60: 22–23, Plate 1, Fig. 18–19. [“Île de la Table, baie d’Along”].
Systenostoma defixa – Pilsbry 1917: Manual of Conchology…: 226, Plate 38, Figs 15–16.

##### Material examined.

*Systenostoma defixa* (Bavay & Dautzenberg, 1912) (2 specimens), RBINS Dautzenberg Collection reg. nr. IG 10591 (tray 844), probably syntypes.

##### Remarks.

The whole shell, including the protoconch, is covered with regular, very fine spiral threads. The spiral lines are decussated with rather irregular radial lines, creating a reticulated surface.

**Figure 1.**
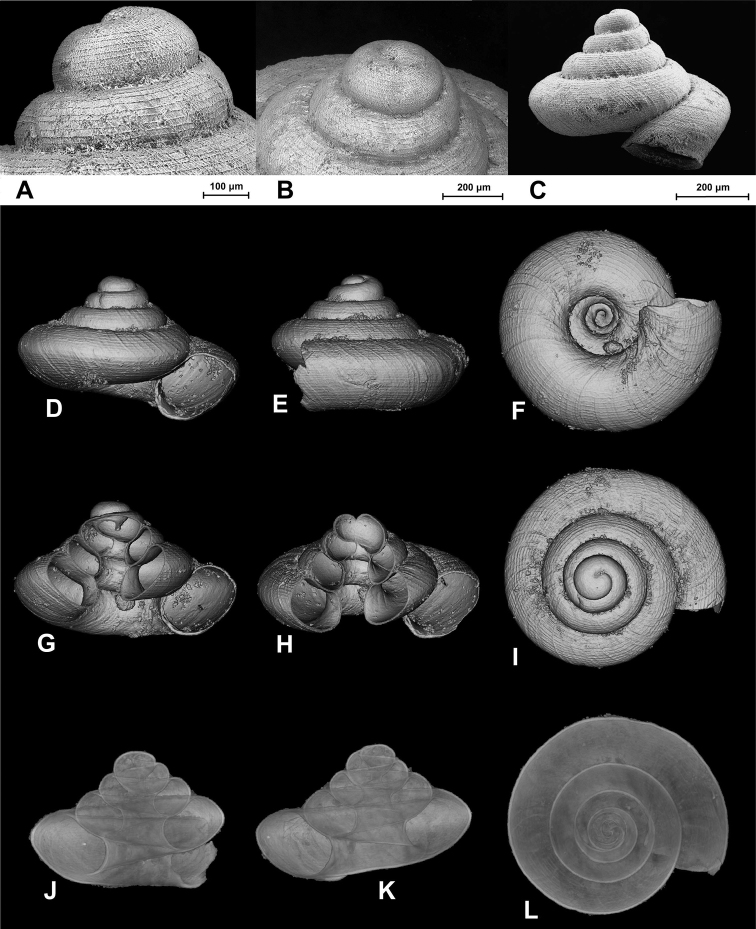
SEM (**A–C**) and Nano-CT Volume Compositing 3D (**D–I**) and Nano-CT Summed Voxel Projection images (**J–L**) of *Tonkinospira defixa* (Bavay & Dautzenberg, 1912). RBINS Dautzenberg Collection reg. nr. IG 10591 (tray 844). Type locality material. Photos: SEM: Suzanne Leidenroth (SMNS). Nano-CT: Gunhild Martels.

#### *Tonkinospira depressa* (Jaeckel 1950)

*Systenostoma depressa* Jaeckel 1950: Archiv für Molluskenkunde, 79: 15–16, Plate 1, Fig. 1. [from river debris, no type locality specified].

#### Tonkinospira pauperrima

(Bavay & Dautzenberg, 1908)

http://species-id.net/wiki/Tonkinospira_pauperrima

Figure 2

Helix (Systenostoma) pauperrima Bavay & Dautzenberg, 1908: Journal de Conchyliologie, 56: 243–244. [“Phu-Quoc-Oaï”].
Helix (Systenostoma) pauperrima – Bavay and Dautzenberg 1909: Journal de Conchyliologie, 57: 195–196, Plate 8, Fig. 4–6. [“Trouvé à Phu-Quoc-Oaï, parmi les détritus coquilliers”]
Systenostoma pauperrima – Pilsbry 1917: Manual of Conchology…: 225–226, Plate 38, Figs 3–5.
Systenostoma pauperrima – Schileyko *Ruthenica* 1998: Supplement 2(2): 165, Fig. 202.

##### Material examined.

*Helix (Systenostoma) pauperrima* (Bavay & Dautzenberg, 1908) (1 specimen). RBINS Dautzenberg Collection reg. nr. IG 10591 (tray 844), probably syntype.

##### Remarks.

The whole shell, including the protoconch is covered with regular, extremely fine spiral threads. The number of threads increases from the apex towards the aperture. The spiral lines are decussated with irregular radial lines, resulting in in a reticulated surface.

**Figure 2.**
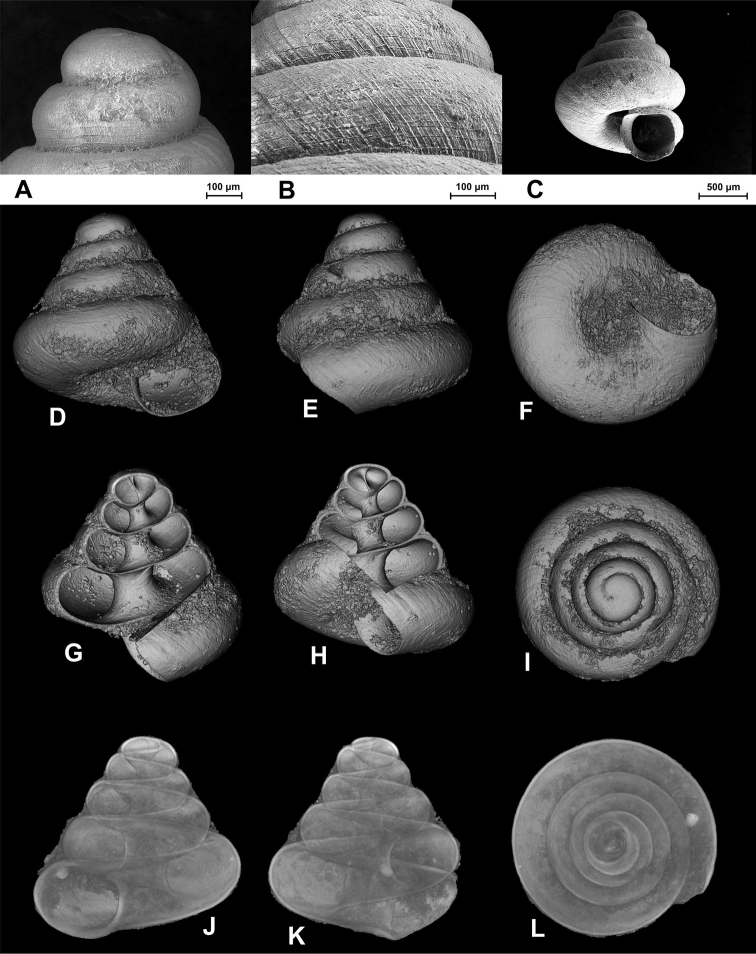
SEM (**A–C**) and Nano-CT Volume Compositing 3D (**D–I**) and Nano-CT Summed Voxel Projection images (**J–L**) of *Tonkinospira pauperrima* (Bavay & Dautzenberg, 1908). RBINS Dautzenberg Collection reg. nr. IG 10591 (tray 844). Type locality material. Photos: SEM: Suzanne Leidenroth (SMNS). Nano-CT: Gunhild Martels.

#### Tonkinospira pulverea

(Bavay & Dautzenberg, 1908)

http://species-id.net/wiki/Tonkinospira_pulverea

Figure 3

Helix (Systenostoma) pulverea Bavay & Dautzenberg, 1908: Journal de Conchyliologie, 56: 243. [“Phu-Quoc-Oai”].
Helix (Systenostoma) pulverea – Bavay and Dautzenberg 1909: Journal de Conchyliologie, 57: 194–195, Plate 8, Fig. 7–9. [“Vit sur les rochers de Phu-Quoc-Oaï”]
Systenostoma pulverea – Pilsbry 1917: Manual of Conchology…: 225, Plate 38, Figs 10–12.

##### Material examined.

*Helix (Systenostoma) pulverea* Bavay & Dautzenberg, 1908 (1 specimen), RBINS Dautzenberg Collection reg. nr. IG 10591 (tray 844), probably syntype.

##### Remarks.

The teleoconch shows rather regular, very fine spiral threads. These spiral lines are more numerous than in the other two examined species. The spiral lines are decussated. Irregular, impressed varices occur at intervals across the whorls. At higher magnification, the shell shows a highly flocculent texture.

**Figure 3.**
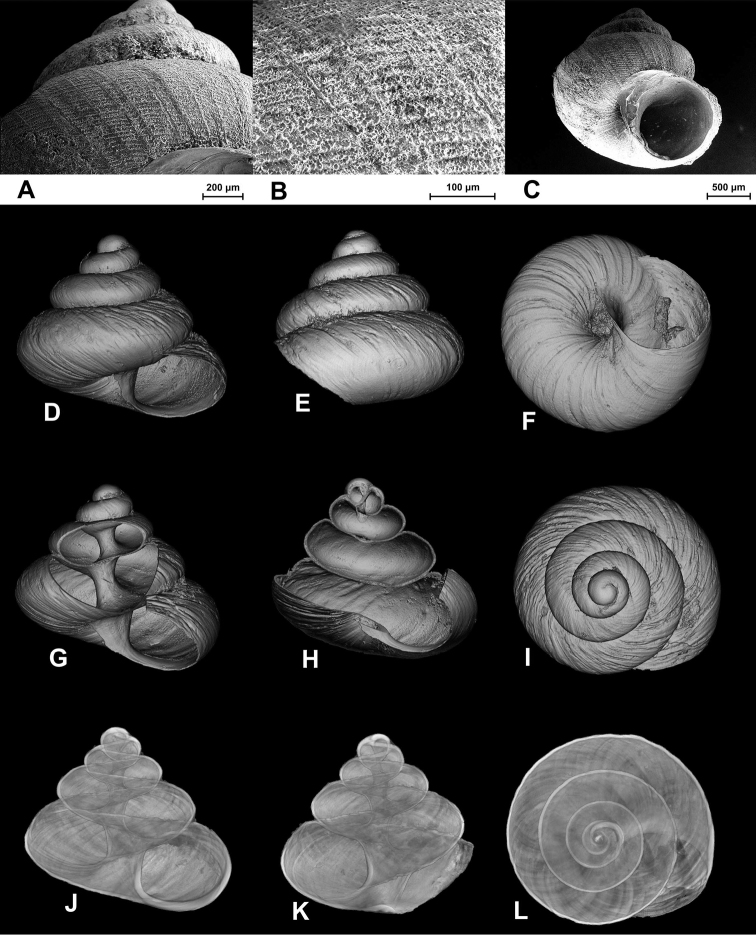
SEM (**A–C**) and Nano-CT Volume Compositing 3D (**D–I**) and Nano-CT Summed Voxel Projection images (**J–L**) of *Tonkinospira pulverea* (Bavay & Dautzenberg, 1908). RBINS Dautzenberg Collection reg. nr. IG 10591 (tray 844). Type locality material. Photos: SEM: Suzanne Leidenroth (SMNS). Nano-CT: Gunhild Martels.

**Figure 4.**
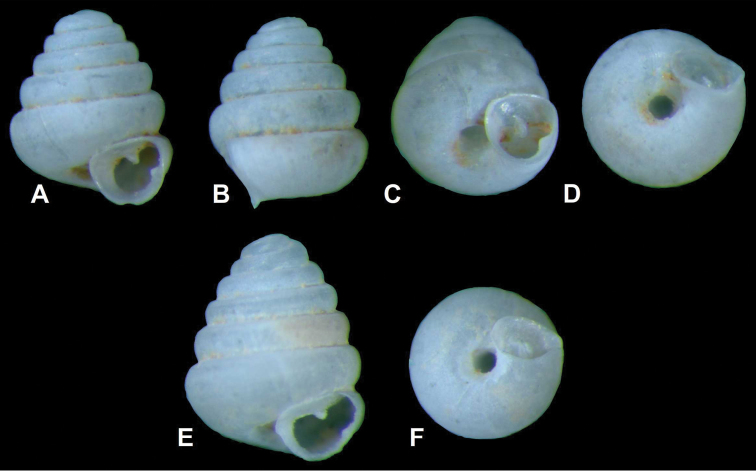
Holotype (**A–D**) and paratype (**E–F**) specimens of *Angustopila huoyani* sp. n. China, Hunan (湖南省), Xiangxi (湘西土家族苗族自治州), Longshan (龙山县), Huoyan (火焰), Feihu Dong (飞虎洞), (ca. 29°12.53'N, 109°18.37'E), soil, leg. Verovnik, 13.04.1997. Photos: Sigrid Hof (SMF).

**Figure 5.**
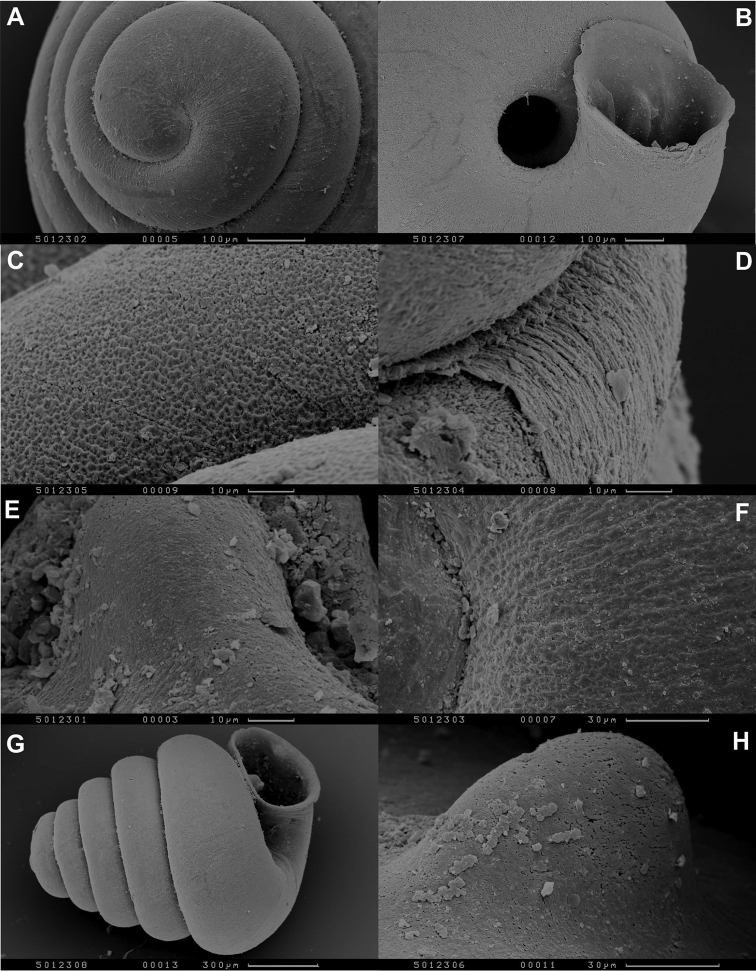
SEM images of *Angustopila huoyani* sp. n. paratype. Same data as in Fig. 4. **A** protoconch **B** umbilicus and adnate aperture **C** reticulating microgranules on whorls **D** fine axial lamellae **E** palatal denticle **F** reticulation on protoconch **G** shell profile **H** parietal callus. Photos: Yaron Malkowsky (SMF).

**Figure 6.**
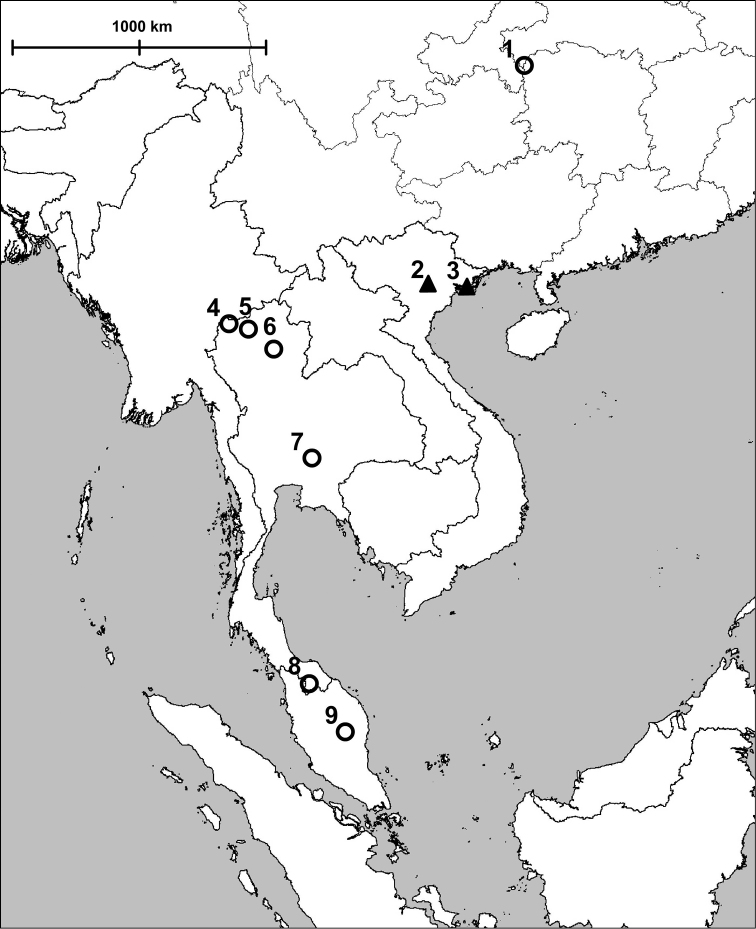
Map showing the localities of *Angustopila* gen. n. (empty circle) *Tonkinospira* nom. n. (filled triangle) and species. **1** *Angustopila huoyani* sp. n. **2** *Tonkinospira pauperrima* (Bavay & Dautzenberg, 1908) and *Tonkinospira pulverea* (Bavay & Dautzenberg, 1908) **3** *Tonkinospira defixa* (Bavay & Dautzenberg, 1912) **4** *Angustopila tamlod* (Panha & Burch, 1999) **5** *Angustopila elevata* (Thompson & Upatham, 1997) **6** *Angustopila edentata* (Panha & Burch, 1999) **7** *Angustopila concava* (Thompson & Upatham, 1997) **8, 9** *Angustopila neglecta* (van Benthem-Jutting, 1961).

## Supplementary Material

XML Treatment for Angustopila

XML Treatment for Angustopila huoyani

XML Treatment for Angustopila neglecta

XML Treatment for Hypselostoma

XML Treatment for Tonkinospira

XML Treatment for Tonkinospira defixa

XML Treatment for Tonkinospira pauperrima

XML Treatment for Tonkinospira pulverea
